# Identification of Factors for the Development of Medical Tourism in the World

**DOI:** 10.3390/ijerph182111205

**Published:** 2021-10-25

**Authors:** Viktoriia Vovk, Lyudmila Beztelesna, Olha Pliashko

**Affiliations:** 1 Department of Economics, Stanisław Staszic University of Applied Sciences in Piła, 64-920 Piła, Poland; 2 Department of Management, Academic and Research Institute of Economics and Management, National University of Water and Environmental Engineering, 33028 Rivne, Ukraine; l.i.beztelesna@nuwm.edu.ua; 3 Department of Economics and Business Management, Faculty of Documentary Communications, Management, Technology and Physics, Rivne State University of Humanities, 33000 Rivne, Ukraine; olga.plyashko@rshu.edu.ua

**Keywords:** medical tourism, international tourism, economic growth, healthcare service, healthcare system, institutional environment

## Abstract

The overall objective of the given paper was to study the relationship of inbound medical tourism destinations with international tourism, economic development of recipient countries, the development of national healthcare systems and the institutional features of their environment, in terms of protection of the rights and freedoms of both business and citizens. In order to achieve this objective, the authors used methods of grouping, as well as correlation and regression analysis. The conducted study revealed that the formation of medical tourism destinations in countries with high social and economic development occurs in a balanced and unidirectional manner; simultaneously, one can see that the countries with “new economic development” form a sufficiently powerful and competitive market for medical tourism. All these countries have one thing in common: namely, there is a link between medical tourism and healthcare funding, international tourism and development of political and civil freedoms. Nevertheless, the noted aspects are not dominant enough, and this indicates that there are other internal factors and their configurations which shape a positive image of countries for medical tourism development. This finding leads to the necessity of further analysis in this field with a breakdown into separate countries or destinations.

## 1. Introduction

The history of medical tourism is very ancient. Most ancient civilizations recognized the therapeutic effect of mineral thermal springs and sacred baths, which provoked travel [1]. Nonetheless, with each passing year, more and more people are interested in overseas medical and health services. Longing to improve their health, people increasingly travel abroad in the hunt for medical care and spend a significant share of their savings. On the other hand, the comprehensive satisfaction of the needs of the medical tourist during his/her visit to the destination country allows countries to receive significant budget revenues, while developing new markets for tourism services.

Medical tourism, like any other type of tourism, is undoubtedly an important component of a steady-state economy. It can contribute to economic diversification and increase of the country’s profitability not only through the inflow of foreign currency, but also by increasing employment of local people, improving the skills of local staff, stimulating investment in healthcare, improving the quality of medical and associated services, as well as improving the health of the country’s own nation. According to Simpson L., people can travel from developed to developing countries and vice versa. In the first case, tourists search for cheaper medical services when there are viable technologies. In the second case, they search for services that are unavailable or are of an unsatisfactory quality in their country [1]. It is no wonder that the competition for attracting tourists in the global market is constantly growing.

In recent decades, medical tourism, as a branch of the tourism economy [2], has been growing quite actively. Patients, especially from developed countries, are increasingly seeking medical care across national borders. People’s desire to be constantly aware of their health, as well as to be in the trend of a healthy lifestyle, encourages not only timely treatment, but also regular health procedures and preventive medical examinations. Therapeutic, rehabilitative and preventive procedures provided by sanatoriums, prevention and treatment facilities and polyclinics facilitate the recovery of patients with chronic cardiovascular, orthopedic, rheumatologic and neurological diseases. Besides, the above procedures attract young people who pay great attention to a healthy lifestyle and physical fitness [3]. Such aspirations, combined with the ease of travel between countries, have led to the growth of medical tourism as a growing industry in many countries [4]. The rise of medical tourism emphasizes the privatization of health care, the growing dependence on technology, uneven access to health resources and the accelerated globalization of both health care and tourism [5].

According to the World Tourism Organization, there has recently been a steady trend of tourism growth in all directions. Throughout 2009–2018, the number of trips related to visiting friends and relatives, medical treatment and improving health, as well as visits for religious reasons increased from 252.32 million people to 377.67 million people [6].

International tourism in the context of travel has always included an economic basis, which was merely consigned to travelers’ purchases of various goods and services [7,8,9], including medical ones. In the latter case, the purpose of such purchases for the person consuming the service was to attain health or subjective well-being [10].

Medical tourism, as such, has existed since ancient times [11]; however thanks to the processes of globalization, it has become widespread. The reasons for this growth are the following: the cost of treatment in wealthy countries, the long queues for certain types of medical services (especially in cases of surgery), the availability of better technologies, practitioners and paramedical staff abroad, the greater variety of medical institutions and methods and treatments, inadequate or completely absent health insurance, the need to maintain anonymity in treatment, the unavailability (prohibition) of necessary treatment at home (e.g., for ethical reasons), the comparable availability of air fares in combination with the availability of direct connections and contributory exchange rates between the countries, etc. [5,11,12,13,14,15].

The emergence of tour operators, intermediaries between international patients and healthcare networks, which offer various packages of medical services for different types of budgets, also contributes to a significant intensification of medical tourism development. Their main mission is to provide information on the destination, with a detailed description of the quality of healthcare and the surrounding infrastructure [16].

The process of planning a trip is due, in no small part, to associated services, which arise mainly due to the accompaniment of tourists by members of their families. Therefore, together with medical tourism growth, other sectors of the economy actively develop at the same time, which leads to the fact that the state treasury shall be filled with foreign currency [17,18].

- The interests of a medical tourist largely coincide with the interests of an international tourist; however, despite the common features, medical tourism has its own features: within the framework of medical tourism, the pleasure of traveling arises not only during the trip itself, but also after the return of the tourist to his/her home, when the patient can feel the long-term improvement of his/her health;
- Traveling in pairs is often a necessity, which can be caused by the desire to feel safer during treatment and to receive additional supervision and assistance in unforeseen situations;
- Usually, medical tourists are characterized by higher incomes and qualifications than ordinary international tourists, and, as a rule, such trips are initiated by women [19], who, unlike men, are more prone to such trips.

These differences arise primarily due to other motivations of medical tourists. The key motives of medical tourism include the following:

1. Commercial factors (lower cost of treatment and diagnosis in developing countries; combination of medical tourism with traditional tourism, which is the priority patient);
2. Qualitative factors (more modern level of medical technologies, expectations of more qualified medical care and service, focus on the medical achievements of the selected doctor) [20];
3. Social factors (lack of paid health insurance in their country, presence of health insurance that does not cover the disease, lack of full state health insurance combined with high cost of private health insurance, unavailable procedures, i.e., “bypass tourism”, or procedures that are not provided in their country) [11,15,21,22];
4. Personal factors (the need to maintain confidentiality, the bias of the patient’s views, previous personal experience or the experience of people in the circle of trust, i.e., intimate circle).

It is clear that the study of medical tourism should be comprehensive, as it is integrated into the economic, social, cultural, personnel and local structure. In the economic literature, there are numerous studies on the interaction of medical tourism with economic growth [23,24,25,26,27]. They revealed that this type of tourism can contribute to economic diversification and profitability by increasing employment, ensuring the provision of healthcare facilities with material resources and improving capital turnover. There are also additional bonuses; as such, a country can count on increasing demand for medical services in the domestic market, as well as improving public health, while this process, in the long run, will once again have a positive impact on economic development [28].

Having studied the specific functioning of modern medical systems concerning the dynamics of healthcare spending in BRICS countries (Brazil, Russia, India, China, and South Africa), scientists from the United States, India, Russia, and Serbia highlighted the economic benefits of healthcare. Besides, they pointed out that the bold gains in the living standard and purchasing power of citizens gives momentum for all of the BRICS to increase investment in health care, far more than majority of nations worldwide [29]. Three clear patterns were also established as follows: (1) the significant increase in the share of Brazil, Russia, India, China and South Africa in global health expenditures from 1995 to 2013; (2) the strong dominance of Chinese national spending among the BRICS countries studied; (3) the long-term trend of increasing the share of global health spending of the BRICS compared to the leading industrialized G7 countries.

The effectiveness of health systems was the subject of a cross-regional comparison of health reform outcomes in Southeastern Europe between 1989 and 2012. Using macroeconomic indicators, researchers found significant differences in health resources and system outcomes of three policy legacies (post-Semashko Eastern European countries, the former Yugoslavia and free-market countries before 1989). Through the evaluation of selected indicators of health system capacity and resource availability, it was illustrated that, despite the different historical legacies of each group of countries under study, they were able to increase life expectancy, ensure better survival of newborns and reduce the number of hospitalized patients. At the same time, these different paths to common goals created a golden opportunity for these economies to learn from each other [30]. While recognizing the importance of medical tourism for the entire economy, the overall objective of the given research was to identify the impact of economic development factors of states, their institutional environment and the performance of national healthcare systems on the formation and development of progressive tendencies for medical tourism. Pursuing this objective gives an opportunity to identify which factors are decisive for the development of medical tourism in recipient countries. This part, in turn, will shape strategic priorities for the development of national healthcare systems based on the partnership between businesses and the state, allowing a country to actively compete in the medical tourism market, while forming a positive image of oneself in the international arena and increasing one’s own well-being. Hence, the hypothesis of our study was that the formation and development of inbound medical tourism destinations is associated with international tourism and economic development of recipient countries, the development of national healthcare systems and the institutional features of their environment, in terms of protection of the rights and freedoms of both business and citizens.

The given paper is structured as follows. Based on the literature review, the main section presents the specifics of the functioning of medical tourism and the views of scientists on the factors of intensification of medical tourism development, which allowed the authors to form a research hypothesis. The next section describes the methodological aspects of the research and provides the sources of the research materials. The final section closes with the results of the research and conclusions.

## 2. Materials and Methods

In order to test the hypothesis, the given study chose the rating value of the countries, according to the Medical Tourism Index (MTI), developed by the International Health Research Center, as an evaluation indicator of medical tourism development. The importance of studying the impact of this indicator is determined by the fact that this indicator specifically gauges any consumer interest and may be used in future MTIs to focus on some of the most sought-after target markets [31].

Particularly for the research purposes, the countries for which information is given in the MTI were selected. It was found that it is reasonable to choose the Medical Tourism Index for 2016, as not all of the studied indicators had data available for a later date. Some of the countries included in the MTI ranking (United Arab Emirates, Taiwan and Korea) were not included in our study. The reason for this is that on other indicators, the data regarding these countries are summarized (for instance, there are aggregated indicators for the United Arab Emirates, without division into the emirates of Dubai and Abu Dhabi) or absent (in case of Korea and Taiwan), which makes conducting qualitative research and comparison impossible. Thus, the study included 37 countries out of the 41 included in the 2016 MTI rankings.

The economic development of countries was assessed with the use of the GDP per capita. The evaluation of international tourism was carried out according to the indicators of the Travel and Tourism Competitiveness Index (TTCI), the number of international tourist arrivals, revenues from international tourism and the increase in tourism investments in 2016 compared to 2015 (Appendix A Table A1).

The development of national health care systems was assessed with the use of current expenditure on health per capita in US dollars (total and state), domestic general government expenditure on health per capita in US dollars, the share of private and out-of-pocket health expenditures and the number of hospital beds and doctors/physicians per 1000 people (Table A2).

Aiming to assess the institutional environment of countries that are medical tourism destinations, we analyzed international competitiveness indices (their absolute values) of the noted states, namely the Global Competitiveness Index (GCI), Human Development Index (HDI), Corruption Perceptions Index (CPI), Doing Business (DB) index, the International Property Right Index (IPRI), the Index of Economic Freedom (IEF), Political Rights Index (PRI) and Civil Liberties Index (CLI) (Table A3).

The research used the subsequent information databases, and this allowed collecting, summarizing and processing the following indicators:

1. The report on Medical Tourism Index–Global Destination [32] for Medical Tourism Indices;
2. The Travel & Tourism Competitiveness Reports [33] for the indicators of the Travel and Tourism Competitiveness Index;
3. The World Data Atlas [34] for the indicators on international tourism, arrivals and incomes from international tourism, investment growth, GDP per capita, current expenditure on health per capita, domestic general government expenditure on health per capita, private health expenditures per capita, out-of-pocket expenditure, doctors per 1000 people and hospital beds per 1000 population;
4. Official reports on international indices for indicators such as the Global Competitiveness Index [35], Human Development Index [36], Corruption Perceptions Index [37], as well as for indicators such as Doing Business, the International Property Right Index, the Index of Economic Freedom, Political Rights Index and Civil Liberties Index [34].

In order to determine the foremost mega-destinations for medical tourism, we used the method of grouping by geographical feature. Aiming to assess the economic development of countries, national healthcare systems and the institutional environment, we developed a matrix of grouping countries by appropriate indicators (with the presentation of above and below average), based on their absolute value compared to the arithmetic mean of the whole. We used the Microsoft Excel software toolkit to create a scatter plot for countries, according to the studied indicators. Conversely, for establishing cause-and-effect links between MTI and international tourism indicators, we used a correlation and regression analysis.

## 3. Results and Discussion

**Figure 1:**
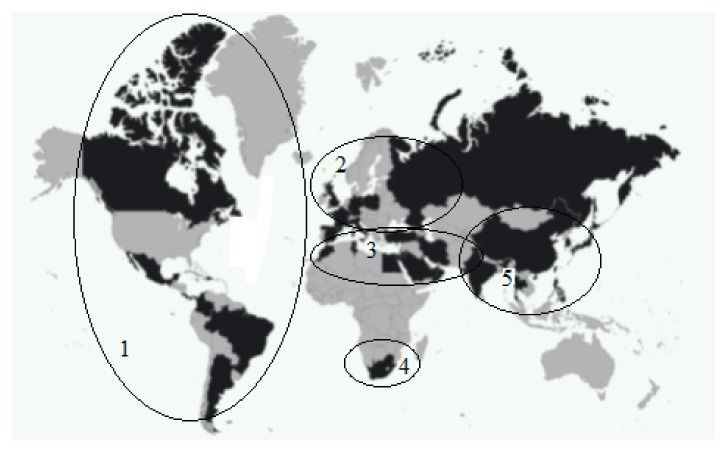
Mega-destinations for Medical Tourism According to MTI, 2016. Legend: 1—American region, 2—European region, 3—Arab and Middle Eastern region, 4—African region, 5—Asian region.

The Medical Tourism Association has developed the conceptual model of the Medical Tourism Index (MTI), and it takes into account environmental factors (economy, security, image, culture), the medical tourism industry (destination attractiveness and medical tourism costs) and the quality of care (quality of healthcare services, reputation of physicians/doctors and healthcare institutions, international accreditations and satisfaction of patients with the quality of medical care and service). According to this study, the foremost medical tourism mega-destinations are as follows (Figure 1): (1) the American region (Canada, Colombia, Costa Rica, Panama, Jamaica, Brazil, Argentina, Mexico, etc.), with its dominant areas of dentistry and cosmetic surgery mainly for U.S. residents; (2) the European region, with countries specializing mainly in the treatment of cancer, cardiology and orthopedic diseases (Great Britain, France, Germany, Italy, Spain, Poland, Russia); (3) the Arab and Middle Eastern region (Bahrain, Egypt, Iran, Israel, Jordan, Kuwait, Lebanon, Morocco, Tunisia, Turkey, Said Arabia, Turkey, Qatar, etc.), with countries specializing in the treatment of oncological diseases, reproductive medicine, cosmetic and plastic surgery and eye microsurgery; (4) African (South Africa and Republic), with countries specializing in the treatment of oncological diseases, reproductive medicine, cosmetic and plastic surgery and eye microsurgery and Asian regions (China, India, Japan, Singapore, Thailand, Korea), with its well-developed practices of general medical examinations and alternative medicine, radical and cosmetic surgery (including sex reassignment) and cancer treatment.

**Figure 2:**
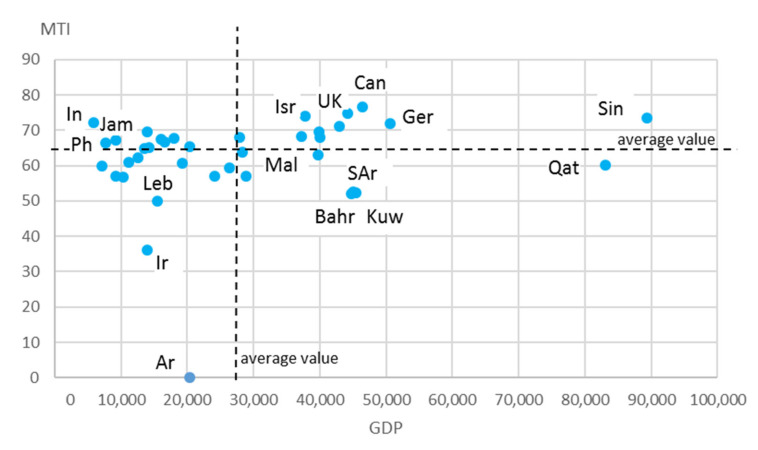
Scattering of Countries According to MTI and GDP per capita. Symbols of countries: Ar—Argentina, Bahr—Bahrain, Can—Canada, Ger—Germany, In—India, Ir—Iran, Isr—Israel, Jam—Jamaica, Kuw—Kuwait, Leb—Lebanon, Mal—Malta, Ph—Philippines, Qat—Qatar, SAr—Saudi Arabia, Sin—Singapore, UK—United Kingdom.

Top medical tourism countries by total rating worldwide included Canada (overall MTI score: 76.62), Great Britain (overall MTI score: 74.87), Israel (overall MTI score: 73.91), Singapore (overall MTI score: 73.56) and India (overall MTI score: 72.1). The countries that had the lowest positions in the MTI rankings included Iran (overall MTI score: 36), Lebanon (overall MTI score: 49.92), Bahrain (overall MTI score: 51.99), Saudi Arabia (overall MTI score: 52.43) and Kuwait (overall MTI score: 52.69). At the same time, for most countries, there was no stable relationship between the country’s MTI ranking and the level of their economic development in terms of GDP (Figure 2).

The countries with a relatively high level of GDP per capita did not necessarily occupy leading positions in the MTI ranking (e.g., Bahrain, Saudi Arabia, Qatar and Kuwait). At the same time, low GDP was not associated with a low MTI ranking (e.g., India, Philippines and Jamaica). There was also no correlation between these indicators (the correlation coefficient was 0.214123).

**Figure 3:**
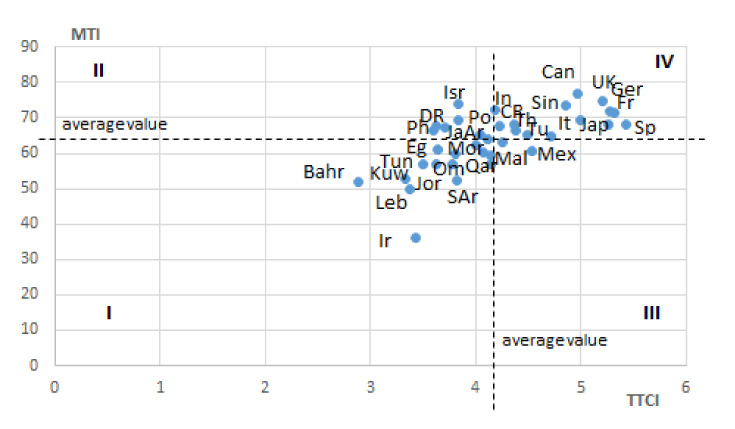
Scattering of Countries According to MTI and TTCI Indicators. Symbols of countries: Ar—Argentina, Bahr—Bahrain, Ca—Canada, CR—Costa Rica, DR—Dominican Repub-lic, Eg—Egypt, Fr—France, Ger—Germany, In—India, Ir—Iran, Isr—Israel, It—Italy, Ja –Jamaica, Jap—Japan, Jor—Jordan, Kuw—Kuwait, Leb—Lebanon, Mal—Malta, Mex—Mexico, Mor—Morocco, Om—Oman, Ph—Philippines, Po—Poland, Qat—Qatar, SAr—Saudi Arabia, Sin—Singapore, Sp –Spain, Th—Thailand, Tun—Tunisia, Tu—Turkey, UK—United Kingdom.

While tracing the links between the MTI rankings and the Travel and Tourism Competitiveness Index, TTCI, one can see that they are somewhat different (Figure 3). Most countries with a high TTCI rating (above average) had top ranking positions for the MTI (4th quadrant: Spain, Canada, Singapore, Great Britain, Germany, France, etc.). Similarly, most countries with a low TTCI level had low positions (below average) according to the MTI (1st quadrant: Bahrain, Kuwait, Iran, Lebanon, Kuwait, etc.). These are mainly countries in the Arab and Middle Eastern regions, with relatively high GDP levels.

In the context of the studied relationship, there were specific features in the cases of the countries with a low TTCI ranking (below average) and high MTI (2nd quadrant: countries of the American region, like Dominican Republic, Jamaica, Philippines, Argentina, etc. and Israel), as well as a high TTCI and low MTI (3rd quadrant: Mexico and Malta). There was a correlation between the MTI and TTCI indicators; however, its parameters (correlation coefficient was equal to 0.66201, R^2^ = 0.4383, *p* <0.05) did not indicate its cause-and-effect (causal) nature.

Given the absolute characteristics of international tourism, we could identify the following groups of countries: countries with a high level of both medical and international tourism development (India, Spain, Japan, Jamaica, Philippines, Panama and Singapore) and countries with a high medical tourism ranking and below average international tourism indicators (Canada, Great Britain, Germany, Israel, Colombia, Thailand, Argentina, Brazil, China and Israel). These groupings allowed us to make assumptions about the target orientation of medical tourists to these countries, as well as to Arab countries (Kuwait, Saudi Arabia, Iran and Oman), where the international tourism indicators were higher, while medical tourism indicators were below average, which most likely indicates the formation of relatively new destinations for medical tourism (Table 1).

The presence of correlations (Table 2) between the indicators studied above, as well, indicates the existence of a link between medical and international tourism. Nevertheless, the further regression analysis (Table A1) for acceptable parameters of the model showed a statistically significant relationship with only one indicator: international tourism revenue (here, we considered incoming international tourism arrivals). The coefficient of elasticity for this indicator (E = 0.107) indicated the insignificant role of incoming cash flows from international tourism in the formation of the MTI ranking, as an increase in revenues from international tourism by 1%, in a certain set of countries, may increase the ranking MTI value by 0.11%. However, such dependencies may be different in cases of different countries.

Based on the assumption that medical tourism development is associated with the development of national healthcare systems, we studied the links between medical tourism and (1) indicators of health care resources in the studied countries (number of hospital beds and doctors/physicians per 1000 people) and (2) the amount and sources of funding for the healthcare system. We did not reveal any connections in the first direction, and hence, we can state that the internal (domestic) system of public healthcare services and medical tourism are separate directions of medical activity. However, in the second direction, the results were somewhat different. Accordingly, countries with relatively high healthcare expenditures (above average) and a predominantly public funding occupied high positions in the medical tourism ranking (Germany, Canada, Japan, France, Great Britain, Israel, Italy, Spain), whereas a high share of private expenditures with minor state and total healthcare funding provided a low ranking of the countries in terms of medical tourism (India, Philippines, Egypt, Morocco, Tunisia and China) (Table 3). At the same time, within the framework of the study, the countries of so-called “ultra-modern development” appeared to be very peculiar. For instance, we could observe Qatar (the MTI ranking was lower than the average, while there were high total and public expenditures on healthcare and a low share of private ones), Singapore (high medical tourism ranking; both public and private expenditures were high), Malta (average medical tourism ranking, high total and public expenditures and low private but high out-of-pocket healthcare expenditures).

Nonetheless, we have found no stable correlation and regression relationships between these indicators, which again confirms the separate positioning and development of the internal healthcare system and exports of healthcare services (at least for the vast majority of the studied countries).

**Figure 4:**
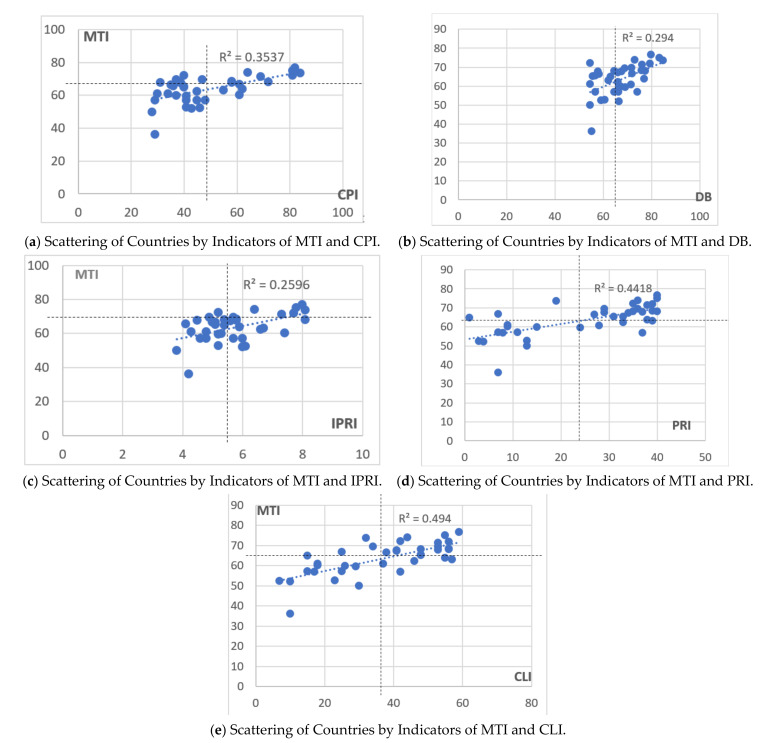
Scattering of Countries by Indicators of MTI and (**a**) international Corruption Perceptions Indices (CPI), (**b**) Doing Business (DB), (**c**) the International Property Right Indices (IPRI), (**d**) Political Rights Indices (PRI) and (**e**) Civil Liberties Indices (CLI).

A correlation analysis of IMD and international indices characterizing the institutional environment for the protection of rights and freedoms of both business and citizens revealed a significant relationship between MTI and the Corruption Perceptions Index (CPI, correlation coefficient was r = 0.594), Doing Business index (DB, correlation coefficient was r = 0.542), the International Property Right Index (IPRI, correlation coefficient was r = 0.509), Political Rights Index (PRI, correlation coefficient was r = 0.664) and Civil Liberties Index (CLI, correlation coefficient was r = 0.703). The relationship between medical tourism and the Political Rights Index (PRI) and Civil Liberties Index (CLI) was the closest to the normal distribution, and it was also tangible. However, these dependencies cannot be considered causal due to the low value of the coefficient of determination, R^2^. Figure 4 shows the scatter plots without identifying the countries, since the relationships between the indices were statistically insignificant. Thus, the scatter plots illustrate the general characteristics of such relationships.

The grouping of the countries according to the ranking values of the studied indices revealed the following features: the MTI’s top-ranked and bottom-ranked countries occupied similar positions in other rankings. The top countries were the United Kingdom, Germany, Canada and Japan (these countries were in the top 10 for all rankings); the bottom-ranked countries were Iran, Lebanon and Saudi Arabia (except for the International Property Right Index). Israel, Italy, Spain and Poland occupied high ranking positions (above average). At the same time, within the framework of the study, some countries appeared to be very peculiar. Particularly, these included Singapore (high ranking positions in all ratings, except for the Political Rights Index (PRI) and Civil Liberties Index (CLI)); India had the 5th place in the medical tourism ranking and the last ranking in Doing Business index; it also ranked low in Corruption Perceptions Indices (CPI) and had higher than average ranking positions in the indices of political rights and civil liberties; Malta had high and medium rankings in all indices except Doing Business; Colombia had a high ranking for MTI (9th place) and Doing Business and ranked below average for all other indicators.

## 4. Conclusions

The conducted research revealed that various factors have an influence upon the formation of medical tourism destinations in different countries. In the cases of countries with high social and economic development (European countries, Canada, Japan), both environment and tourism, including the medical one, develop in a balanced and unidirectional way, which contributes to the formation of resources for further development. The countries of so-called “ultra-modern development” (Asian, Arab and Middle Eastern countries) are different in terms of social and institutional characteristics, as well as regarding sources of funding for the domestic healthcare system; however, they also form a sufficiently powerful and competitive market for medical tourism. Nevertheless, all countries have some features in common as well. Particularly, there is a link between medical tourism and healthcare funding, international tourism and the development of political and civil liberties, but the cause-and-effect (causal) links between them do not exist, or they are not significant enough (as in the case of revenues from international tourism). This fact indicates the presence of other, internal factors and their configurations, which form a positive image of countries for medical tourism development and, hence, substantiates further analysis in this direction in terms of individual countries or destinations.

Each country has its own model of healthcare system functioning, which is the basis for forming export medical services, i.e., the development of medical tourism. However, the structure of financing the national models is similar. In turn, medical tourism development is caused not only by the healthcare system evolving, but by infrastructural and institutional factors formed under the influence of national socio-economic policy and government cooperation with businesses and households under financing activities.

In order to have a successful medical tourism industry, it is necessary to create conditions for tourists that would meet or even exceed their expectations, while creating more benefits for locals than costs [38]. This should be linked to the country’s macroeconomic policy through properly chosen fiscal and monetary policies, as well as proper regulation of the labor market [39] and foreign economic performance of the state.

It is also worth noting that the COVID-19 pandemic challenges have significantly affected both the functioning of national healthcare systems and the development of medical tourism [40]. Therefore, it seems relevant and promising to further investigate the established characteristics in the years of the Medical Tourism Index formation and find common features in the context of countries and their associations, as well as to develop scenarios for the further development of medical tourism.

## Figures and Tables

**Table 1:** Matrix of Grouping Countries by Medical and International Tourism Indicators.

The Value of Indicators	Medical Tourism Index (MTI)	International Tourist Arrivals	International Tourism Revenue	Investment Growth
Above average	Canada United Kingdom Israel Singapore India Germany France Italy Colombia Spain Japan Panama Costa Rica Dominican Republic Jamaica Thailand Philippines Argentina Brazil China Poland	Spain *Kuwait* India Jamaica *Iran* Japan *Malta* Jordan *Saudi Arabia* Lebanon Philippines	*Kuwait* Spain Japan *Saudi Arabia* *Iran* India Jamaica Philippines Lebanon Panama *Malta* Oman Singapore	India Panama Spain Philippines Japan Russia *Iran* Lebanon Israel Singapore *Kuwait* Oman Jamaica *Malta* *Saudi Arabia*
Below average	*Malta* South Africa Egypt Mexico Qatar Morocco Turkey Jordan Russia Oman Tunisia *Kuwait* Saudi Arabia Bahrain Lebanon *Iran*	Russia Singapore Morocco Panama Oman South Africa United Kingdom Tunisia Brazil Canada Israel Qatar Italy Bahrain Colombia Costa Rica Argentina Germany Turkey France Dominican Republic Egypt Thailand Poland Mexico China	Russia Jordan Turkey Morocco Tunisia South Africa China Italy Israel Dominican Republic Qatar Mexico Germany Canada Argentina United Kingdom Costa Rica France Colombia Thailand Egypt Bahrain Poland Brazil	Canada Jordan Tunisia Colombia South Africa Dominican Republic Costa Rica Morocco Germany Qatar Turkey China Mexico Bahrain Argentina Egypt Italy France Thailand Brazil United Kingdom Poland

For the underlined countries all the studied indicators are above average (except Singapore). Countries in italics have above-average value of MTI and below-average value of other studied indicators.

**Table 2:** Results of Correlation Analysis for MTI and International Tourism Indicators.

Indicators	MTI	International Tourist Arrivals	Income from International Tourism	Investment Growth
MTI	1			
International tourism, arrivals	0.756775	1		
Incoming International tourism arrivals	0.777265	0.97488	1	
Investment growth	0.584937	0.858542	0.811382	1

**Table 3:** Matrix of Grouping Countries by Medical Tourism and Healthcare Funding Indicators.

The Value of Indicators	Medical Tourism Index	Current Expenditure on Health per Capita	Domestic General Government Expenditure on Health per Capita	Private Health Expenditures per Capita	Out-of-Pocket Health Expenditure
Above average	Canada United Kingdom Israel Singapore India Germany France Italy Colombia Spain Japan Panama Costa Rica Dominican Republic Jamaica Thailand Philippines Argentina Brazil China Poland	Germany Canada France Japan United Kingdom Israel Italy Singapore Spain Malta Qatar	Germany Japan Canada France United Kingdom Italy Israel Spain Qatar Malta Singapore	Bahrain China Russia Tunisia South Africa Jordan Mexico Iran Lebanon Singapore Dominican Republic Brazil Morocco Philippines Egypt India	Brazil Panama Bahrain Jordan Singapore Lebanon Malta China Tunisia Russia Mexico Iran Dominican Republic Philippines Morocco Egypt India
Below average	Malta South Africa Egypt Mexico Qatar Morocco Turkey Jordan Russia Oman Tunisia Kuwait Saudi Arabia Bahrain Lebanon Iran	Saudi Arabia Bahrain Kuwait Panama Argentina Costa Rica Poland Brazil Lebanon Oman Mexico Russia Turkey Iran South Africa Colombia Dominican Republic China Jordan Jamaica Tunisia Thailand Morocco Egypt Philippines India	Kuwait Saudi Arabia Argentina Panama Bahrain Costa Rica Oman Poland Turkey Brazil Lebanon Colombia Russia Mexico China Iran South Africa Dominican Republic Jamaica Thailand Jordan Tunisia Morocco Egypt Philippines India	Oman Japan Kuwait Qatar United Kingdom Turkey Germany France Thailand Italy Argentina Canada Costa Rica Spain Poland Colombia Saudi Arabia Panama Israel Jamaica Malta	Oman South Africa Qatar France Thailand Japan Germany Canada United Kingdom Saudi Arabia Argentina Kuwait Colombia Turkey Jamaica Costa Rica Israel Italy Poland Spain

## Data Availability

The data used in this study are publicly available on the following websites: https://www.unwto.org/global-and-regional-tourism-performance (accessed on 28 July 2012); https://www.medicaltourism.com/destination-healthcare-guide (accessed on 28 July 2012); https://www.weforum.org/reports/the-travel-tourism-competitiveness-report-2017 (accessed on 28 July 2012); https://knoema.com/atlas (accessed on 28 July 2012); http://www3.weforum.org/docs/GCR2016-2017/05FullReport/TheGlobalCompetitivenessReport2016-2017_FINAL.pdf (accessed on 28 July 2012); http://hdr.undp.org/en/content/human-development-report-2016 (accessed on 28 July 2012); https://images.transparencycdn.org/images/2016_CPIReport_EN.pdf (accessed on 28 July 2021).

## Appendix A

**Table A1:** Baseline Data for Assessing Economic Development and International Tourism Development.

Country	Arbitrary Symbol	Travel and Tourism Competitiveness Index	International Tourism, Arrivals, 1000 People	International Tourism Incoming, mln.USD	Investment Growth, %	GDP per Capita
Argentina	Ar	4.05	6668	5466	6	20,307.9
Bahrain	Bahr	2.89	10,158	4021	0.3	44,790.1
Brazil	Braz	4.49	6547	6613	17.3	14,256.2
Canada	Ca	4.97	17,971	18,144	16.1	46,480.47
China	Ch	4.72	59,270	44,432	134.6	13,572.62
Colombia	Col	3.83	3254	5584	2.4	13,952.14
Costa Rica	CR	4.22	2925	3776	0.4	18,008.6
Dominican Republic	DR	3.62	5959	6720	0.6	16,109.86
Egypt	Eg	3.64	5258	3306	4.5	11,192.37
France	Fr	5.32	82,682	63,557	37.6	42,920.27
Germany	Ge	5.28	35,555	52,229	24.5	50,564.25
India	In	4.18	14,570	23,111	37.9	5839.9
Iran	Ir	3.43	4942	3914	2.8	14,011.55
Israel	Isr	3.84	2900	6587	3	37,843.98
Italy	It	4.99	52,372	42,423	9.9	39,922.91
Jamaica	Jam	3.71	2182	2539	0.4	9193.3
Japan	Ja	5.26	24,040	27,285	36	39,970.68
Jordan	Jor	3.63	3567	4943	0.7	9283.871
Kuwait	Kuw	3.33	7055	831	0.4	44,985.35
Lebanon	Leb	3.37	1688	7373	1.2	15,487.11
Malta	Mal	4.25	1966	1451	0.3	39,699.61
Mexico	Mex	4.54		20,619	8.4	19,314.26
Morocco	Mor	3.81	10,332	7481	4.4	7112.99
Oman	Om	3.78	2335	2390	0.7	28,926.14
Panama	Pa	4.37	1921	6280	1.2	27,828.52
Philippines	Ph	3.6	5967	6289	1.8	7703.75
Poland	Pol	4.11	17,471	12,052	2.6	28,283.7
Qatar	Qa	4.08	2938	12,593	1.5	83,102.49
Russia	Ru	4.15	33,729	12,822	5.4	24,125.4
Saudi Arabia	Sar	3.82	18,044	13,438	23.8	45,485.66
Singapore	Sin	4.85	12,913	18,944	15.2	89,386.08
South Africa	Saf	4.01	10,044	8807	4.8	12,592.49
Spain	Sp	5.43	75,315	66,982	15.9	37,282.44
Thailand	Th	4.38	32,530	48,459	7.1	16,618.76
Tunisia	Tun	3.5	5724	1706	0.8	10,359.3
Turkey	Tur	4.14	30,289	26,788	21.4	26,329.36
United Kingdom	UK	5.2	35,814	47,777	23.6	44,162.55

**Table A2:** Baseline Data for Assessing the Development of National Healthcare Systems.

Country	Arbitrary Symbol	Current Expenditure on Health per Capita, US dollars	Domestic General Government Expenditure on Health per Capita, US Dollars	Private Health Expenditures per Capita, US Dollars	Out-of-Pocket Expenditure, Current Expend.%	Doctors per 1000 People	Hospital Beds per 1000 Population
Argentina	Ar	959	711	1311	15.98	4	4.95
Bahrain	Bahr	1099	675	1179	27.99	0.9	1.89
Brazil	Braz	769	342	1077	27.41	2.2	2.11
Canada	Ca	4518	3339	1041	14.13	2.3	2.6
China	Ch	398	231	985	35.91	1.9	2.12
Colombia	Col	419	288	864	16.37	2	1.68
Costa Rica	CR	887	650	785	22.21	1.4	1.15
Dominican Republic	DR	414	189	694	44.62	1.6	1.44
Egypt	Eg	151	47	691	59.01	0.8	1.43
France	Fr	4257	3272	667	9.55	3.2	6.06
Germany	Ge	4734	3657	427	12.88	4.2	8.06
India	In	61	16	424	63.21	0.8	0.48
Iran	Ir	454	230	388	42.29	1.1	1.7
Israel	Isr	2856	1815	358	22.33	3.5	2.99
Italy	It	2736	2042	335	22.93	4	3.17
Jamaica	Jam	280	179	309	17.95	0.5	1.78
Japan	Ja	4175	3508	253	12.83	2.4	13.11
Jordan	Jor	297	147	248	30.1	1.4	1.4
Kuwait	Kuw	1073	901	237	16.04	2.6	2.04
Lebanon	Leb	667	332	226	33.53	2	2.73
Malta	Mal	2328	1464	225	35.02	2.9	4.67
Mexico	Mex	475	249	224	40.52	2.3	1
Morocco	Mor	153	65	202	54.32	0.7	1.1
Oman	Om	645	573	198	6.12	1.9	1.49
Panama	Pa	1040	682	172	27.43	1.6	2.23
Philippines	Ph	130	41	167	53.69	0.6	0.3
Poland	Pol	813	560	150	23.08	2.4	6.64
Qatar	Qa	1800	1491	131	7.95	2.7	1.2
Russia	Ru	469	267	112	40.48	4	8.16
Saudi Arabia	Sar	1166	778	104	15.71	2.4	2.23
Singapore	Sin	2490	1179	101	32.66	2.3	2.48
South Africa	Saf	428	230	101	7.75	0.8	0.3
Spain	Sp	2391	1700	89	23.94	3.8	2.97
Thailand	Th	225	171	88	11.35	0.4	0.3
Tunisia	Tun	257	145	72	39.9	1.3	2.18
Turkey	Tur	469	368	54	16.47	1.8	2.75
United Kingdom	UK	3945	3160	45	15.49	2.8	2.57

**Table A3:** Baseline Data for Assessing Institutional Environment.

Country	MTI	GCI	HDI	CPI	DB	IPRI	IEF	PRI	CLI
Argentina	65.37	3.81	0.827	36	56.73	4.1	43.8	31	48
Bahrain	51.99	4.47	0.824	43	66.64	6	74.3	4	10
Brazil	65.22	4.06	0.754	40	55.62	5.1	56.5	33	48
Canada	76.62	5.27	0.92	82	79.76	8	78	40	59
China	64.78	4.95	0.738	40	63.12	5.4	52	1	15
Colombia	69.48	4.3	0.727	37	68.86	4.9	70.8	29	34
Costa Rica	67.67	4.41	0.776	58	67.67	5.8	67.4	37	53
Dominican Republic	67.58	3.94	0.722	31	57.9	4.5	61	29	41
Egypt	60.92	3.67	0.691	34	54.7	4.3	56	9	18
France	71.22	5.2	0.897	69	76.15	7.3	62.3	38	53
Germany	71.9	5.57	0.926	81	79.5	7.7	74.4	39	56
India	72.1	4.52	0.624	40	54.52	5.2	56.2	35	42
Iran	36	4.12	0.774	29	55.38	4.2	43.5	7	10
Israel	73.91	5.18	0.899	64	73	6.4	70.7	36	44
Italy	69.5	4.5	0.887	47	71.69	5.7	61.2	36	53
Jamaica	67.17	4.13	0.73	39	66.19	5.6	67.4	34	41
Japan	68	5.48	0.903	72	77.53	8.1	73.1	40	56
Jordan	57.02	4.28	0.741	48	56.74	5.7	68.3	11	25
Kuwait	52.69	4.53	0.8	41	60.66	5.2	62.7	13	23
Lebanon	49.92	3.84	0.763	28	54.71	3.8	59.5	13	30
Malta	62.97	4.52	0.856	55	62.28	6.7	66.7	39	57
Mexico	60.7	4.41	0.762	30	71.64	4.8	65.2	28	37
Morocco	59.77	4.2	0.647	37	67.4	5.3	61.3	15	26
Oman	56.9	4.28	0.796	45	66.34	6	67.1	8	17
Panama	67.93	4.51	0.788	38	64.73	5.4	64.8	35	48
Philippines	66.4	4.36	0.682	35	58.23	5.1	63.1	27	38
Poland	63.79	4.56	0.855	62	76.93	5.9	69.3	38	55
Qatar	60.07	5.23	0.856	61	66.49	7.4	70.7	9	18
Russia	57.01	4.51	0.804	29	74.1	4.6	50.6	7	15
Saudi Arabia	52.43	4.84	0.847	46	59.16	6.1	62.1	3	7
Singapore	73.56	5.72	0.925	84	84.89	8.1	87.8	19	32
South Africa	62.2	4.47	0.666	45	66.24	6.6	61.9	33	46
Spain	68.29	4.68	0.884	58	75.93	5.8	68.5	39	56
Thailand	66.6	4.64	0.74	61	71.94	5	63.9	7	25
Tunisia	56.78	3.92	0.725	41	64.57	4.8	57.6	37	42
Turkey	59.49	4.39	0.767	41	69.14	5.2	62.1	24	29
United Kingdom	74.87	5.49	0.909	81	83.34	7.8	76.4	40	55

Legend: MTI—Medical Tourism Index; GCI—Global Competitiveness Index; HDI—Human Development Index; CPI—Corruption Perceptions Index; DB—Doing Business; IPRI—International Property Right Index; IEF—Index of Economic Freedom; PRI—Political Rights Index; CLI—Civil Liberties Index.

**Table A4:** Stepwise Regression Analysis of the Impact of Changes in International Tourism Indicators (x_1_–x_3_) on the Ranking Value of the Medical Tourism Index MTI (y).

Resulting Indicator (y)	Impact Factors (x)	Equation	Parameters			
			Correlation Coefficient, R	Coefficient of Determination, R^2^	*F* Fact, (*p* = 0.95)	|*t*-Fact|, (*p* = 0.95)
MTI	x_1_– International tourism, arrivals x_2_– Incoming international tourism arrivals x_3_– Investment growth	x = 57.21 + 8.83 × 10^−^^5^x_1_+ + 0.00031x_2_ − 0.06x_3_	0.7823	0.6120	*F* crit = 2.8588 17.3547	*t*-crit = 2.03 x_1_ = 0.39 x_2_ = 1.41 x_3_ = 0.82
Disapplication of factor x_1_						
MTI	x_2_– Incoming international tourism arrivals x_3_– Investment growth	x = 57.33 + 0.00039x_2_ − 0.0473x_3_	0.7812	0.6103	*F* crit = 3.2519 *F* fact = 26.5187	*t*-crit = 2.0301 x_2_ = 4.8362 x_3_ = 0.7306
Elasticity coefficient, Ex_2_ = $0.00039*\frac{{\bar{x}}_{2}}{\;y\;¯}\;$ = 0.00039 ∗ $\;\frac{17506.27}{63.48}$ = 0.1079						
